# A reproducible ensemble machine learning approach to forecast dengue outbreaks

**DOI:** 10.1038/s41598-024-52796-9

**Published:** 2024-02-15

**Authors:** Alessandro Sebastianelli, Dario Spiller, Raquel Carmo, James Wheeler, Artur Nowakowski, Ludmilla Viana Jacobson, Dohyung Kim, Hanoch Barlevi, Zoraya El Raiss Cordero, Felipe J Colón-González, Rachel Lowe, Silvia Liberata Ullo, Rochelle Schneider

**Affiliations:** 1 Engineering Department, University of Sannio, Benevento, Italy; 2 School of Aerospace Engineering, Sapienza University of Rome, Rome, Italy; 3 European Space Agency, Φ-lab, Frascati, Italy; 4 Faculty of Geodesy and Cartography, Warsaw University of Technology, Warsaw, Poland; 5 Statistics Department, Fluminense Federal University, Niterói, Brazil; 6 UNICEF, New York, NY USA; 7 Wellcome Trust, Data for Science and Health, London, UK; 8 Centre on Climate Change and Planetary Health and Centre for Mathematical Modelling of Infectious Diseases, London School of Hygiene and Tropical Medicine, London, UK; 9 Tyndall Centre for Climate Change Research, School of Environmental Sciences, University of East Anglia, Norwich, UK; 10 Barcelona Supercomputing Center (BSC), Barcelona, Spain; 11 Catalan Institution for Research and Advanced Studies (ICREA), Barcelona, Spain

**Keywords:** Disease prevention, Public health

## Abstract

Dengue fever, a prevalent and rapidly spreading arboviral disease, poses substantial public health and economic challenges in tropical and sub-tropical regions worldwide. Predicting infectious disease outbreaks on a countrywide scale is complex due to spatiotemporal variations in dengue incidence across administrative areas. To address this, we propose a machine learning ensemble model for forecasting the dengue incidence rate (DIR) in Brazil, with a focus on the population under 19 years old. The model integrates spatial and temporal information, providing one-month-ahead DIR estimates at the state level. Comparative analyses with a dummy model and ablation studies demonstrate the ensemble model’s qualitative and quantitative efficacy across the 27 Brazilian Federal Units. Furthermore, we showcase the transferability of this approach to Peru, another Latin American country with differing epidemiological characteristics. This timely forecast system can aid local governments in implementing targeted control measures. The study advances climate services for health by identifying factors triggering dengue outbreaks in Brazil and Peru, emphasizing collaborative efforts with intergovernmental organizations and public health institutions. The innovation lies not only in the algorithms themselves but in their application to a domain marked by data scarcity and operational scalability challenges. We bridge the gap by integrating well-curated ground data with advanced analytical methods, addressing a significant deficiency in current practices. The successful transfer of the model to Peru and its consistent performance during the 2019 outbreak in Brazil showcase its scalability and practical application. While acknowledging limitations in handling extreme values, especially in regions with low DIR, our approach excels where accurate predictions are critical. The study not only contributes to advancing DIR forecasting but also represents a paradigm shift in integrating advanced analytics into public health operational frameworks. This work, driven by a collaborative spirit involving intergovernmental organizations and public health institutions, sets a precedent for interdisciplinary collaboration in addressing global health challenges. It not only enhances our understanding of factors triggering dengue outbreaks but also serves as a template for the effective implementation of advanced analytical methods in public health.

## Introduction

Dengue is a vector-borne disease spread between humans by *Aedes aegypti* and *Aedes albopictus* mosquitoes^1^. More than half of the world population is exposed to the risk of morbidity with estimated 10,000 deaths per year^2^. Dengue affects children directly through a spectrum of clinical disease and complications, but also indirectly by affecting their relatives^3,4^. Dengue is endemic tropical and sub-tropical regions, where specific environmental conditions favour transmission (i.e. humidity, temperature, rainfalls), and especially affects densely populated areas, such as large urban centres^5,6^. In the future, it is expected that population at risk of dengue will increase mainly due to two factors: climate change and urbanisation. Climate change will be responsible not only for increasing the transmission in already affected regions, but also for expanding the geographical extent of the disease, as warmer temperatures will span over more months per year at high latitudes and altitudes, including current dengue-free regions in Europe, Asia, North America and Australia^2,7^. Urbanisation can also significantly contribute to the growing population at risk of dengue as a densely populated area with associated large mosquito populations provides the ideal environment for maintenance of the viruses and the periodic generation of epidemic strains^8^. Dengue has been identified as one of the most important emerging tropical diseases^9^, which has mobilised both local initiatives^5^ and global agendas to reduce mortality and morbidily associated with the disease (e.g. the Sustainable Development Goals by United Nations within 3.3 target for neglected tropical diseases)^10^.

As vaccine development is still a work in progress and currently no antiviral drugs are available, the prevention measures primarily rely on reducing the risk of human-mosquito contacts^6^. In particular, these measures include direct means, such as fans, mosquito coils, insecticides sprays, screen windows, professional pest control^11^ and indirect activities, e.g., removing discarded water containers, house inspections, etc.^5^. Local and national authorities in the most impacted areas usually have limited resources to implement preventative measures, which leads to a need for targeted campaigns that can act at a time and place where they can be the most effective. Therefore, precise and timely spatial predictions of dengue risk is crucial. It is relevant to note that children are particularly vulnerable to severe dengue, which explains the interest of the United Nations, and UNICEF specifically, in this topic^12,13^.

Containing and modeling Dengue risk encompass a spectrum of challenges rooted in the complex dynamics of the disease. The adaptability of Aedes mosquitoes, primary vectors for Dengue, introduces a significant obstacle. Additionally, the unpredictable interplay of environmental factors, including temperature, humidity, and rainfall, across diverse regions adds layers of complexity to predictive modeling efforts. This diversity makes it challenging to formulate universally applicable models capable of accurately capturing the multifaceted nature of Dengue transmission.

Human mobility emerges as another critical factor complicating Dengue dynamics. Understanding and predicting the movement of people within and between regions pose challenging tasks, particularly in areas characterized by high population mobility. The presence of multiple Dengue serotypes further complicates the scenario, as immunological factors associated with prior infections can significantly impact the severity of subsequent cases. Limited or incomplete data on Dengue cases, mosquito populations, and environmental variables pose additional challenges, hindering the development of robust predictive models.

Furthermore, climate change introduces a dynamic element, influencing the distribution of vectors and disease incidence. Predicting the evolving impact of climate patterns on Dengue dynamics requires sophisticated modeling approaches. Urbanization, especially rapid and unplanned growth, contributes to the complexity by creating conducive environments for Aedes mosquitoes. The dynamic nature of urban landscapes makes it challenging to predict how urban development, population density, and Dengue transmission interact. Finally, the effectiveness of Dengue prevention and control measures is intricately tied to the healthcare infrastructure in place, with limited resources or inadequate healthcare systems in certain regions posing challenges to implementing and sustaining effective control measures. Addressing these multifaceted challenges demands collaborative efforts across disciplines, bringing together expertise from epidemiology, entomology, climatology, and data science, alongside the development of advanced modeling approaches capable of comprehensively capturing the intricate factors influencing Dengue transmission and associated risks.

Various methods devoted to the prediction of dengue incidence have been developed so far. They involve different kinds of modelling strategies, from time series analysis of dengue incidence^14,15^, to more advanced models which incorporate also other variables related to the factors that influence dengue variation, including temperature, humidity and precipitation^16–23^, indicators related to the El Niño Southern Oscillation^9,21^, demographic and socio-economic data^21,22,24,25^, altitude^9,21^, biome, road densities^6^, vegetation-related indices^26^. Some recent attempts also incorporated social media and internet search queries data to predict dengue outbreaks^27,28^. It is worth noting that most previous dengue modelling studies have been developed at either the local or national level. This is mainly due to different definitions, procedures and practices applied when collecting dengue data in different countries, leading to data inconsistency that does not allow for building generalised models capable of predicting dengue incidence across national boundaries.

Several statistic modelling techniques have been applied to model dengue incidence, for example, Autoregressive Integrated Moving Average (ARIMA)^14,19,22^, Seasonal Autoregressive Integrated Moving Average Models (SARIMA)^20^, Integrated Nested Laplace approximations (INLA)^29,30^, various versions of exponential smoothing models^15^, Seasonal Trend Decomposition using Loess (STLM)^15^, and a negative binomial regression^6^. However, many recent studies have applied Machine Learning (ML) methods, which are complex enough to be able of capturing complicated correlations among used variables and to capture the complex dynamics of the disease. ML modelling includes Support Vector Machine (SVM)^16,17^, neural network model (NNETAR)^15^, structural model (StructTS)^15^, Generalised Linear Model or Generalised Linear Mixed Model^9,20^, multilayer perceptron models (MLP)^15,20^, Random Forest^22^ and Long-Short term memory recurrent neural networks (LSTM)^20^. Although most reported approaches uses single ML learners, several studies applied ensemble methods that combine SVM predictors^18^, neural networks, SARIMA and Generalised Linear Model^20^. Several studies in the literature use Latin American countries as case studies to predict dengue risk at different spatio-temporal aggregation levels. For example the Brazilian cities and sub-regions, at yearly^16–18^ and monthly levels^14,20,25^. Furthermore, there are also country-scale analyses resulting in monthly-based predictions^9,15^. Some modelling studies have been developed for Peru^1,21–23^, mainly for the city of Iquitos in the Peruvian Amazon, at the weekly temporal scale.

Based on these consideration, we formulated our research question and subsequently developed a methodology aimed at assisting organizations in preventing the dissemination of Dengue and/or mitigating outbreaks, with a specific emphasis on the well-being of children.

Consequently, in this study, we proposed and applied a novel ensemble approach that leverages the use of different ML methods, including deep neural networks, to predict DIR one month ahead in all 27 Brazilian Federal Units (FU) from 2001 to 2019. We then transferred the trained ML-based ensemble model to predict DIR for several departments in Peru, between the years of 2010 and 2019, where a smaller dataset is available. Our analysis is focused on the entire population with a specific focus on children and youth up to 19 years old, as they represent the most vulnerable group to dengue. Our model is designed to give as output two distinct forecast, one for children/youth (0–19 years old) and one for total population. Children are particularly a vulnerable to the disease because their immune systems are weaker; in endemic areas, children can get dengue at a very early age and have little protection against other serotypes. With a second infection, they are more likely to develop severe dengue.

The main contributions of this paper are summarized as follows, we introduced a pioneering ensemble approach tailored for a unique multi-modal dataset. Our methodology combines both machine-learning and deep-learning models, meticulously configured to handle temporal and multi-modal data effectively. The dataset we present encompasses a diverse array of variables, including eco-climatic, environmental, and population factors, relevant to the spread of Dengue over an extensive time period. Through comprehensive analysis, we highlighted the significance of the multi-modality of our dataset, showcasing a notable performance improvement compared to solutions exclusively focused on Dengue data. Our approach, trained to predict Dengue Incidence Rate (DIR) across Brazil from 2001 to 2019, was successfully validated in a distinct scenario, namely Peru from 2010 to 2019. Notably, our approach excels in examining vulnerability, particularly in individuals aged up to 19 years, offering distinct predictions for this demographic and the general population. The application and integration of the ensemble machine learning into operational frameworks is what constitutes the true innovation of our approach. This work contributes significantly to the advancement of climate services for health, providing a template for how academic research can inform practical applications. Dengue fever, despite being a neglected tropical disease, imposes substantial public health and economic consequences. Our ensemble model offers a timely and effective forecasting system to empower local governments.

The remaining part of the paper is structured as follows. Section "Materials and methods" introduces the methodology proposed and the data used in section "Results" we present the results obtained with the proposed method, tested on different scenarios. In section "Discussion" we discuss the limitations and advantages of our approach. Section "Conclusion" concludes our paper.

## Materials and methods

The DIR forecasting method we employed leverages the power of an ensemble comprising three distinct machine learning models. This ensemble consists of a CatBoost model, a SVM, and a LSTM model. Each of these models possesses unique characteristics that, when combined, demonstrate good performance in predicting the DIR in Brazil and Peru.

The first model in our ensemble is CatBoost, a gradient boosting algorithm that excels in handling categorical features and generating accurate predictions. CatBoost utilizes an ensemble of decision trees to effectively capture complex relationships within the data. The second model in our ensemble is an SVM, a powerful algorithm known for its ability to find optimal hyperplanes in high-dimensional spaces. SVMs excel in handling both linear and nonlinear data and can effectively identify patterns and trends within the DIR data, leading to accurate forecasts. The third model incorporated in our ensemble is an LSTM, which is a type of recurrent neural network (RNN) capable of capturing temporal dependencies in sequential data. LSTMs are particularly suited for time series forecasting tasks, as they can effectively learn from past observations and capture long-term patterns and dynamics. By combining these three models in an ensemble, we take advantage of their unique strengths. The ensemble methodology allows us to leverage the strengths of each model, resulting in a comprehensive and robust forecasting approach.

Through extensive training and optimization, our ensemble of these three models has demonstrated high performance in predicting the DIR one month in advance. By leveraging the peculiarities of each model and combining their predictions, we achieve more accurate and reliable forecasts, enabling proactive measures and interventions to mitigate the impact of dengue outbreaks. To train our DIR forecasting model, we constructed a new dataset, including a diverse range of variables, such as satellite-based products and socio-economic variables, which are grouped into distinct clusters.

The datasets generated and/or analysed during the current study are available in the ESA-UNICEF_DengueForecastProject repository, https://github.com/ESA-PhiLab/ESA-UNICEF_DengueForecastProject.

### Modelling approach

**Figure 1:**
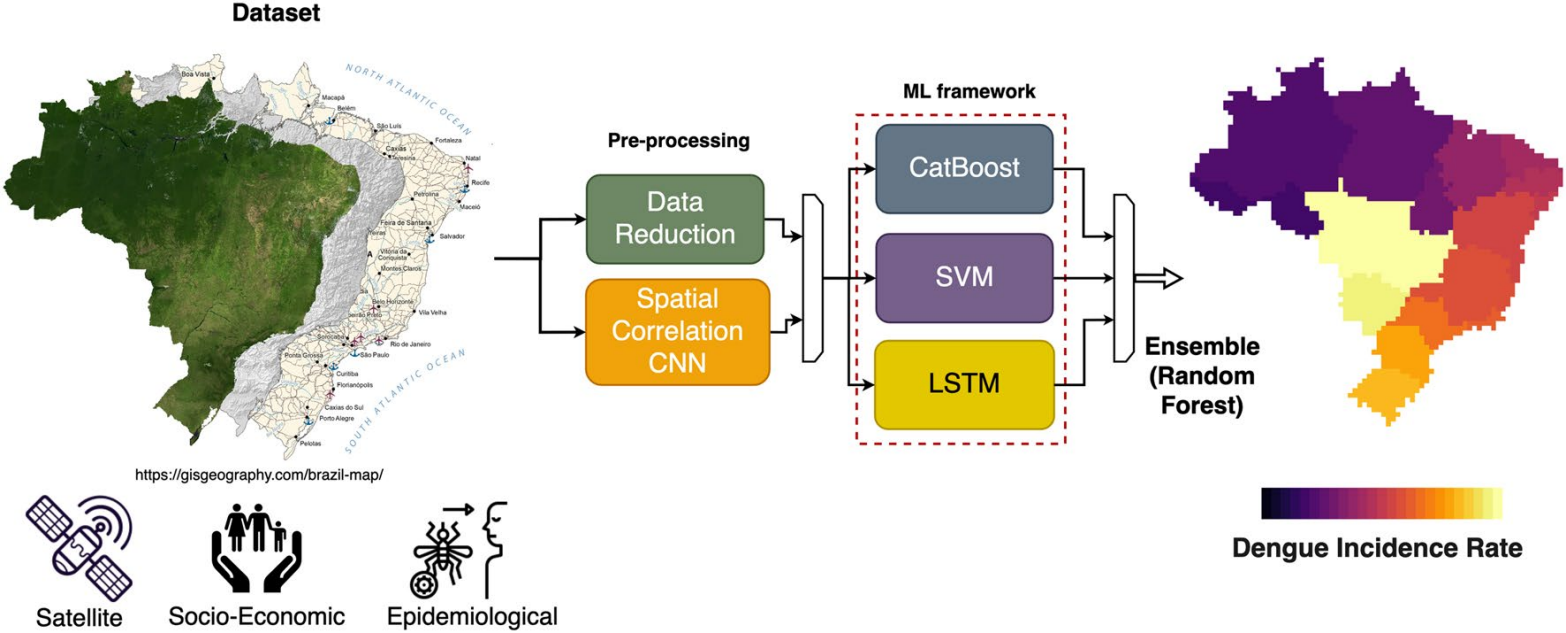
The proposed model for the forecasting of the DIR in Brazil. Starting from left, the dataset is firsly pre-processed by applying data reduction through PLS, then spatial correlation is calculated. After this the new dataset is used to train the ML framework comprising of a CatBoost, an LSTM and an SVM model. The ensable ends with a Random Forest models that combines previous models output to return the final prediction and the confidence interval. The schematic ends with the DIR forecast map over Brazil.

Figure 1 displays the ML model framework, comprising the following ML techniques: (i) Categorical Boosting (CatBoost), (ii) Support Vector Machine (SVM), and (iii) Long Short-Term Memory (LSTM). We fused the results of each ML model by inputting their outputs to a Random Forest model. A key benefit of using an ensemble approach is to improve the average prediction performance over any of the single weak learner in the ensemble^31^. We adopted classic loss functions aiming to produce a high generalised model since sophisticated loss functions can lead to less transferable features^32^. As proof of this concept, in section Transfer learning: Peru we applied our pre-trained ensemble ML model in a different study case (Peru).

*CatBoost* CatBoost is a ML method based on Gradient Boosting (GB) Decision Tree, which uses binary decision trees as base predictors. A decision tree^33,34^ divides the original feature space ${{\mathbb {R}}}^m$ into disjoint areas, also called leaves, with a constant value in each region, according to the values of some splitting attributes. In other words, the result of a decision tree learning is a disjoint union ($\sqcup$) of subsets $\{X_1, X_2,\ldots , X_q : \sqcup _{i=1}^q X_i = {{\mathscr {X}}}\}$ and a piecewise constant function $f({\varvec{x}}) = \sum _{i=1}^q\mathbbm {1}_{\{{\varvec{x}} \in X_i\}}c_i, \,c_i\in {{\mathbb {R}}}$, where $\mathbbm {1}$ is the indicator function. GB is a powerful ML technique as it can solve problems with heterogeneous features, noisy data and complex dependencies, and is effective both on small and big datasets. Further information are already reported in other works^35–38^.

Consider a dataset of *n* examples, $D = \{({\varvec{x}}_k, y_k)\}_{k=1,\dots ,n}$, where ${\varvec{x}}_k = (x^1_k,\dots , x^m_k)\in {{\mathscr {X}}}\subset {{\mathbb {R}}}^m$ is the vector of features and $y_k\in {{\mathbb {R}}}$ is the target value. The goal of the learning task is to train a function $F: {{\mathbb {R}}}^m \rightarrow {{\mathbb {R}}}$ which minimises the expected loss ${{\mathbb {E}}}L(y, F(x))$. A GB procedure iteratively builds a sequence of approximations $F^t: {{\mathbb {R}}}^m \rightarrow {{\mathbb {R}}},\,t = 0, 1, \dots$ using a so-called greedy stagewise approach^39^. Namely, $F^t$ is obtained from the previous approximation $F^{t-1}$ in an additive manner: $F^t = F^{t-1} + \alpha h^t$, where $\alpha$ is a step size and $h^t: {{\mathbb {R}}}^m \rightarrow {{\mathbb {R}}}$ (a base predictor) is chosen from a family of functions *H* in order to minimize the expected loss:

1 $$\begin{aligned} h^t = \mathop {\textrm{argmin}}\limits _{h\in H}{{\mathbb {E}}}L(y, F^{t-1}({\varvec{x}}) + h^t({\varvec{x}})). \end{aligned}$$

For the training of our CatBoost model, we used the Multivariate RMSE as loss function:

2 $$\begin{aligned} {{{\mathscr {L}}}}(\theta ) = \sqrt{\frac{1}{N}\sum _{i=1}^{N} (y(i) - {\hat{y}}_{\theta }(i))^2} \end{aligned}$$

where *N* is the number of data points, *y*(*i*) is the *i*-th measurement, and ${\hat{y}}(i)$ is its corresponding prediction, made with network weights $\theta$.

*SVM* SVMs are a set of supervised learning methods used for regression and classification, first developed by Vapnik et al.^40^. When SVM is applied to a regression problem, it is denominated Support Vector Regression (SVR). Unlike simple linear regression, SVR seeks to minimise the coefficients of a defined loss function, that equally penalises high and low misestimates, while providing flexibility on how much error is acceptable in the model. SVR attempts to find the narrowest $\epsilon$-insensitive region ($\epsilon$-tube) that best approximates the continuous-valued function while minimising the prediction error, in such a way that the absolute values of errors less than a certain threshold $\epsilon$ are ignored both above and below the estimate. In this manner, points outside the $\epsilon$-tube are penalised, while those within the $\epsilon$-tube receive no penalty.

The definition of the margin width $\epsilon$ relies on the data points located outside and closer to the decision boundary, which are called *support vectors*. The latter are the training samples that will influence the model’s prediction.

Both SVM and SVR allow to accommodate non-linear fits to the data by means of a kernel approach^41^. When the classes are not linearly separable, *kernel tricks* are used to map non-linearly separable functions into a higher dimensional space, called kernel space, where a linearly separable function can be applied without ever explicitly computing the transformation of the features in the kernel space. Instead, one simply needs the computation of a generalised inner product formula, given by the kernel, between the input vector and the support vectors, making it a much more computationally efficient process.

The implementation of this model was performed using the scikit-learn library. Multiple parameters have to be set to use SVR: (i) $\epsilon$, defines the maximum error (width of the tube); (ii) *C*, defines the tolerance to margin violations; and (iii) the kernel approach (for instance linear kernel, Radial Basis Function (RBF) or polynomial kernel). The parameter *C* is considered a regularisation parameter in the sense that the higher its value, the less tolerance there will be for margin violations, hence the wider the tube will be, decreasing the number of support vectors and therefore decreasing the regularisation strength (increasing the variance). In this study, we implemented an extensive grid search that yielded the following best hyperparameters: $C=1$, $\epsilon = 0.01$ and a 3*rd*-degree polynomial kernel.

The $\epsilon$-insensitive loss function used to train our SVR model is given by Equation 3:

3 $$\begin{aligned} {{{\mathscr {L}}}}(\theta ) = {\left\{ \begin{array}{ll} \displaystyle \sum _{i=1}^{N} |y(i) - {\hat{y}}_{\theta }(i)|-\varepsilon &{} \hbox { if}\ |y(i) - {\hat{y}}_{\theta }(i)| \ge \varepsilon \\ 0 &{} \text {otherwise} \end{array}\right. } \end{aligned}$$

where $\varepsilon \ge 0$ defines the maximum error, *N* is the number of data points, *y*(*i*) is the *i*-th measurement, and ${\hat{y}}(i)$ is its corresponding prediction, made with network weights $\theta$.

*LSTM* Recurrent Neural Networks (RNN) are a class of neural networks that is powerful for modelling sequence data such as time series or natural language^42^. Differently from the independent and identically distributed input data, elements in a sequence appear in a certain order and are not independent from each other. Schematically, a RNN layer uses a *for loop* to iterate over the time-steps of a sequence, while maintaining an internal state that encodes information about the time-steps it has seen so far.

LSTMs are a special kind of RNN, capable of learning long- and short-term dependencies. They were introduced by Hochreiter and Schmidhuber^43^. LSTMs perform well on a large variety of problems dealing with time dependencies, and are now widely used for time-series analysis, audio classification, video interpretation, etc. All RNNs have the form of a chain of repeating modules of NN. In standard RNNs, this repeating module has a very simple structure, whereas in LSTM is composed of a cell, an input gate, an output gate and a forget gate. The cell remembers values over arbitrary time intervals and the three gates regulate the flow of information into and out of the cell. With respect to RNNs, LSTMs use an extra piece of information, called memory, for each time step in every LSTM cell. The LSTMs are formed by six components: forget gate *f*, candidate layer *c*, input gate *i*, output gate *o*, hidden state *h* and memory state *c*.

The mathematics defining LSTMs is summarized by the equations (4), from which it is possible to note how this network treats temporal data:

4a $$\begin{aligned} f_t&= \sigma (x_t \circ W_{xf} + h_{t-1}\circ W_{hf} + b_f) \end{aligned}$$

4b $$\begin{aligned} {\hat{C}}_t&= tanh(x_t \circ W_{xc} + h_{t-1}\circ W_{hc} + b_c)\end{aligned}$$

4c $$\begin{aligned} i_t&= \sigma (x_t \circ W_{xi} + h_{t-1}\circ W_{hi} + b_i)\end{aligned}$$

4d $$\begin{aligned} o_t&= \sigma (x_t \circ W_{xo} + h_{t-1}\circ W_{ho} + b_o)\end{aligned}$$

4e $$\begin{aligned} C_t&= f_t \circ C_{t-1}+ i_t \circ {\hat{C}}_t\end{aligned}$$

4f $$\begin{aligned} h_t&= o_t \circ tanh(C_t) \end{aligned}$$

where $\circ$ represents the Hadamard product, $\sigma$ represents the sigmoid function $\sigma (x)=\frac{1}{1+e^{-x}}$, $x_t$ is the input vector, $h_{t-1}$ is the previous cell output, $C_{t-1}$ is the previous cell memory, $h_t$ is the current cell output, $C_t$ is the current cell memory, $b_{f/c/i/o}$ are bias coefficients , *W* are the weight vectors for the forget gate, candidate gate, i/p gate, o/p gate and $\omega$ blocks represent the neural network layers.

For the training of our LSTM model we used the MAE as loss function:

5 $$\begin{aligned} {{{\mathscr {L}}}}(\theta ) = \frac{1}{N}\sum _{i=1}^{N} |y(i) - {\hat{y}}_{\theta }(i)| \end{aligned}$$

where *N* is the number of data points, *y*(*i*) is the *i*-th measurement, and ${\hat{y}}(i)$ is its corresponding prediction, made with network weights $\theta$.

*Random Forest*   RF is a supervised learning algorithm that merges multiple bagged decision trees together in order to obtain a single low-variance statistical learning model^44^. To build a RF model in a regression problem, one must build and train *B* regression trees separately, using *B* bootstrapped training datasets. The process of building a single regression tree involves recursively selecting the best predictor to split the predictor space into distinct and non-overlapping regions so as to minimise the residual sum of squares within each of the resulting regions. Then for every observation that falls into region *r*, its prediction is the mean of the response values for the training observations in *r*. The final prediction of the RF model is ultimately computed by averaging the *B* probabilistic predictions from all *B* regression trees. To avoid high correlation between the trees within the forest, RF forces each split of the data to consider only a random subset of the predictors.

Once the RF model has been trained, it is possible to compute the prediction intervals, that is to estimate an interval into which the future observations will fall with a given probability (or confidence level)^45^. In order to achieve this, one simply needs to enforce that each tree in the forest is fully expanded, so that each leaf has exactly one value. Then each prediction returns individual response variables from which a distribution can be built and percentile statistics can be derived. In this use case, we computed prediction intervals with a percentile of 95%. For our Random Forest model training, we used the MSE as loss function:

6 $$\begin{aligned} {{{\mathscr {L}}}}(\theta ) = \frac{1}{N}\sum _{i=1}^{N} (y(i) - {\hat{y}}_{\theta }(i))^2 \end{aligned}$$

where *N* is the number of data points, *y*(*i*) is the *i*-th measurement, and ${\hat{y}}(i)$ is its corresponding prediction, made with network weights $\theta$. The scikit-learn implementation of RF used in this work requires the definition of a few parameters, namely the number of trees, *B*, which was set to 100.

### Machine learning model setup

In this section we present the settings we configured to run the ML models. The main parameters composing our ensemble solution and used to train the models are reported in Table 1.

**Table 1:** Training settings for the adopted models.

Setting	CatBoost	LSTM	SVM	RF
Learning rate	0.001	0.0001	0.001	–
Max. N. Iterations	27,000	200	20,000	–
N. Estimators	–	–	–	100
Max. depth	6	–	–	6
Early stopping	300	12	–	–
Batch size	–	16	–	–
Optimizer	–	rmsprop	–	–

### Dataset description

The dataset is a collection of temporal series of heterogeneous data acquired over the 27 FUs of Brazil, here defined as ${\textbf{X}} \in {{\mathbb {R}}}^{D \times T\times V}$ where *D* represents the FUs, *T* the temporal length of the series, here expressed by 19 years of 12 months, and *V* the heterogeneous variables. To complete the dataset, the sum of DIR has been acquired for the same FUs, during the same temporal interval, and is defined as ${\textbf{Y}} \in {{\mathbb {R}}}^{D \times T}$. The dataset is divided in training and validation by selecting data from 2001 to 2016 for the former and from 2017 to 2019 for the latter.

**Table 2:** List of collected variables and respective source.

Variables	Source	Administrative level	Temporal resolution
Epidemiological and population			
Dengue cases (total)	SINAN^46^	FU	Monthly
Dengue cases age-group (0–19 years)	SINAN^46^	FU	Monthly
Total Population	IBGE^47^	Municipality	2010 (static)
Population age-group (0–19 years)	IBGE^47^	Municipality	2010 (static)
Climatic			
2m Air Temperature (K) − min, mean, max			
2m Dew Point Air Temperature (K)			
Surface Pressure (Pa)	ERA5-Land^48^	Municipality	Daily
Total Precipitation (m)			
10m u/v Wind components (m/s)			
Geophysical			
Normalised Difference Vegetation Index (NDVI)	MODIS^49^	Municipality	Monthly
Elevation (m) − min, mean, max	Shuttle Radar Topography^50^	Municipality	Monthly
Forest Loss / Cover (%)	Landsat 7 and 8^51^	FU	Yearly
Socio-economic			
31 variables (see Appendix 6 or our GitHub repo^52^)	IBGE^47^	FU	2010 (static)

*Data collection and spatio-temporal aggregation*   This study used the largest Brazilian administrative level (i.e. FUs, also known as States) to group monthly dengue cases from January 2001 to December 2019, provided by the Sistema de Informação de Agravos de Notificação (SINAN)^46^. The number of cases by FU was converted into DIR per 100,000 population per state. Population data was obtained on municipality level from the Instituto Brasileiro de Geografia e Estatística (IBGE)^47^ and aggregated to the state level.

Six satellite-based meteorological variables were selected from ERA5-Land global reanalysis dataset provided by the Copernicus Climate Change Service (C3S)^48^ described in Table 2. In addition, the relative humidity was computed using variables from the ERA5 dataset. NDVI was obtained from the NASA Moderate Resolution Imaging Spectroradiometer (MODIS), using a atmospherically and radiometrically corrected product (MOD09GA Version 6)^49^. Regarding spatial and temporal resolutions, the grid cells from all variables were grouped by municipality, through a geometric intersection tool, and averaged monthly from January 2001 to December 2019.

Global Forest Loss product, derived from Landsat 7 and 8 satellites^53^ (version 1.8^51^), provided annual measurements of forest loss by FU. Finally, mean/max/min altitude by municipality were extracted from the digital elevation product from the Shuttle Radar Topography Mission (version 4)^50^.

The subset of variables extracted by municipality (i.e. meteorological variables, NDVI and elevation) were then spatially upscaled to FU level using population-weighted averages, assigning more weight to these environmental variables in heavily populated areas inside each FU. This data was extracted from Google Earth Engine^54^ and was manipulated using Python (https://www.python.org) version 3.7.0.

**Figure 2:**
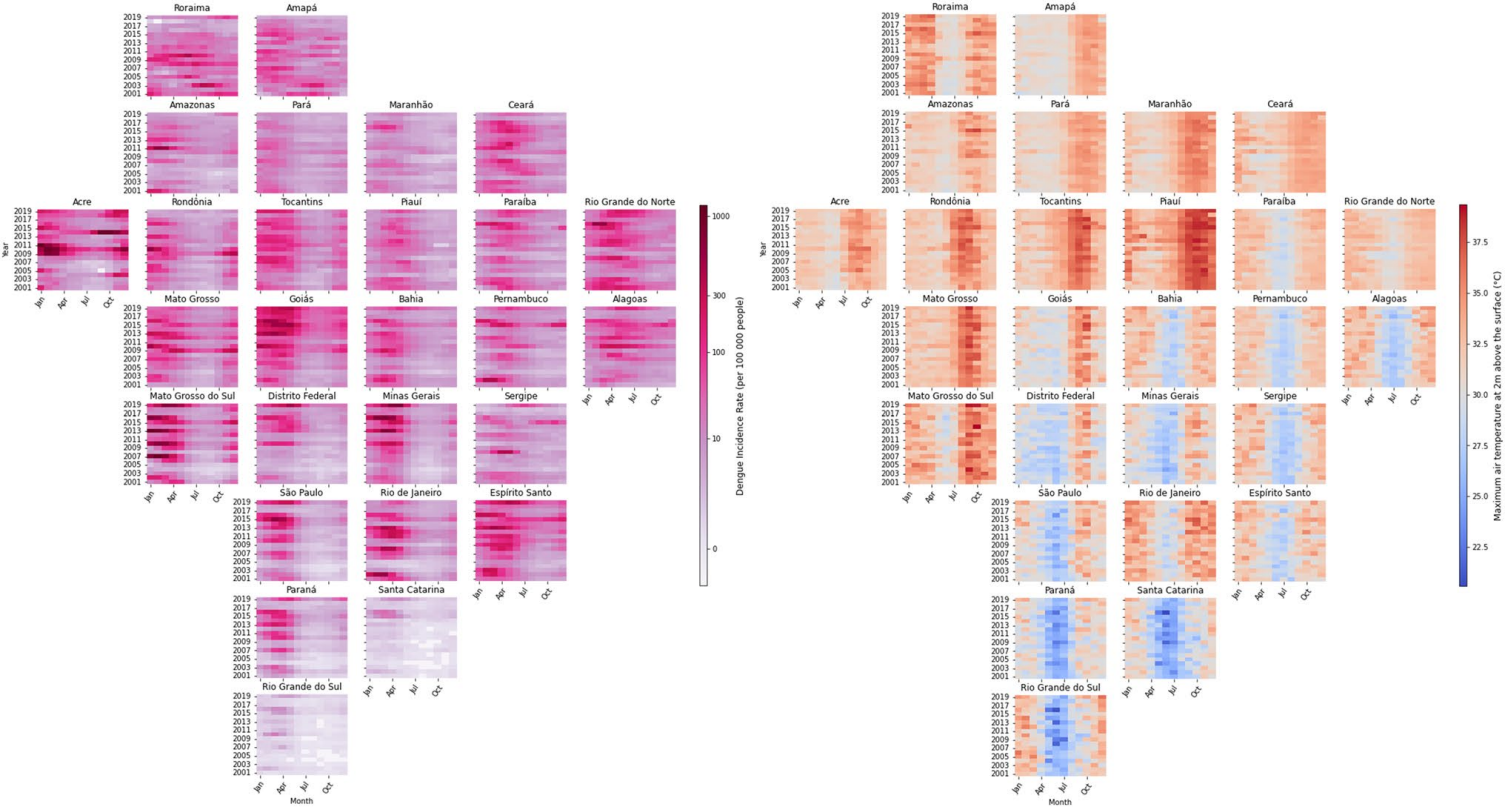
**(Left)** Monthly DIR distribution per 100,000 population for each FU between 2001 and 2019. **(Right)** Monthly population-weighted maximum air temperature at 2m above the surface (^∘^C) for each FU between 2001 and 2019. In both plots, each FU is spatially organised so as to resemble its relative geographic location in Brazil^55,56^.

A graphical visualisation of some variables of the proposed dataset is presented in Fig. 2. Note that the shape of the plot corresponds to the shape of Brazil, where each box represents a FU and illustrates the evolution of the variable in time by setting the x-axis to months and y-axis to years. These plots allow for a comparison between FUs, to visually spot seasonal phenomena or patterns, and they give an immediate interpretation of the variable contained in the dataset. Remaining variables can be found in our GitHub page^52^.

The inclusion of satellite-based products in our dataset enables us to harness the power of remote sensing data. Satellite imagery provides valuable information about environmental factors that can impact the prevalence of dengue. These factors may include temperature, humidity, precipitation, vegetation indices, land cover, and water bodies. By integrating satellite-based products into our dataset, we capture the dynamic and spatial aspects of the environment, enhancing the predictive capabilities of our model. In addition to satellite-based products, we incorporate socio-economic variables into our dataset. Socio-economic factors play a significant role in dengue transmission and incidence rates. Variables such as population density, urbanization levels, housing conditions, access to healthcare facilities, and socio-demographic characteristics contribute to the understanding of the epidemiological patterns of dengue. By including these variables, we capture the socio-economic context in which the disease spreads, allowing for a more comprehensive and accurate forecasting model.

The process of developing this dataset involved thorough research and analysis to identify the most relevant variables and data sources. We collected and processed the data, ensuring its quality and compatibility for training our ensemble of machine learning models. The dataset creation process was iterative, involving continuous refinement and validation to ensure that the selected variables cover a broad spectrum of factors influencing DIRs. By training our ensemble model on our dataset, we leverage the comprehensive nature of the included variables, ranging from satellite-based products to socio-economic factors. This holistic approach allows us to capture the complex dynamics of dengue transmission and generate accurate forecasts, empowering stakeholders and decision-makers to implement targeted interventions and preventive measures.

* Data normalisation* Data normalisation is very common in ML, because it allows the models to learn faster and produce better results^57^. It consists of adjusting the values measured on different scales to a notionally common scale.

Let ${\textbf{x}}^i \in {{\mathbb {R}}}^{D\times T}$ be the *i*-th variable in the dataset ${\textbf{X}}$. Each variable in the dataset has been normalised to the range [0,1], through the min-max scaler of scikit-learn (Python library). The same process was applied to ${\textbf{Y}}$. Note that min and max values have been stored to de-normalise the model prediction to the original scale.

Before applying the ML framework, some dataset pre-processing and data augmentation steps were introduced to account for spatial correlation and to reduce the dimensionality of the data.

**Figure 3:**
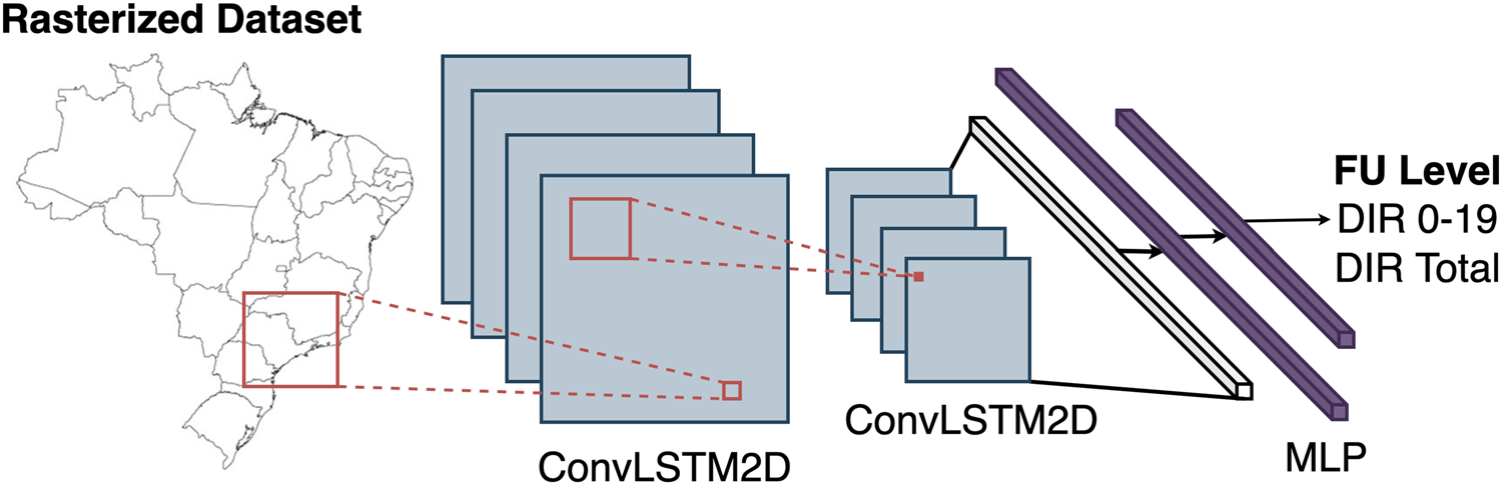
CNN scheme for spatial correlation. ConvLSTM2D layers are used to extract spatial (with Convolution 2D layers and temporal (with LSTM) features. A multilayer perceptron (MLP) uses these features to predict DIR values accounting for spatio-temporal patterns.

* Spatial correlation* An important step of our pre-processing scheme is the addition of the spatial correlation between FUs through a CNN (see Fig. 3). Firstly, we time-ordered and rasterised our FU’s tabular dataset in grid cells with the size of the smallest FU (i.e. Distrito Federal). Then we applied our CNN scheme, composed of three convolutional LSTM 2D layers and a multi-layer perceptron, to forecast DIR values using LSTM architecture but accounting for spatial correlations among FUs. Note that the kernel size and the stride are set to relatively high values, 7 and 3 respectively, to to encourage learning correlations among near states. The CNN-LSTM model has two outputs: (i) DIR total population and (ii) DIR 0–19.

*Dimensionality reduction* In order to simplify the number of variables and types, Partial Least Square (PLS) was applied to the dataset, after the normalisation step, as a dimensionality reduction strategy. By using PLS, the dimension *V* of the dataset was reduced to $V^*$ (e.g. starting from a set of $N=100$ samples, where each sample is a collection of $V=10$ variables, after applying the PLS, by setting the number of output components equals to 4, the set is transformed in a new set containing $N=100$ samples, where each sample is a collection of $V^*=4$ components).

The number of PLS components for each group of variables is 4 for the Climatic group, 6 for the Geophysical one and 10 for the Socio-economical one. It is important to mention that we left the top-10 most important variables out of the PLS, allowing their characteristics to be fully integrated. These variables were chosen based on the variable importance feature available in CatBoost. In this way we allowed the final ensemble to directly learn from the most important variables, while indirectly learning using a reduced version from the long list of variables, maintaining the dataset robust but simplified.

*Data augmentation* After data reduction, the dataset was reshaped to create short time series with the intent of increasing the number of samples for proceeding with the training of the ML framework. This is done by applying a moving window of 12 months with an overlap ratio of $91\%$.

This process results in a new dataset ${\textbf{X}} \in {{\mathbb {R}}}^{N \times D \times T^* \times V^*}$, where *N* is the number of $T^*=12$ time-series obtained by applying the moving window. Moreover, to further increase the size of training data, Gaussian white noise was to the dataset. This process introduced a slight variation of the dataset and by repeating this process $m=3$ times we were able to get the dataset ${\textbf{X}} \in {{\mathbb {R}}}^{(m\times N) \times D \times T^* \times V^*}$.

## Results

### Results on validation

This section presents the main quantitative and qualitative results we obtained using our methodology in Brasil.

First two columns of Table 3 reports the ensemble normalised RMSE (nRMSE) results by FU, for the total population and the 0–19 population groups, respectively.

**Figure 4:**
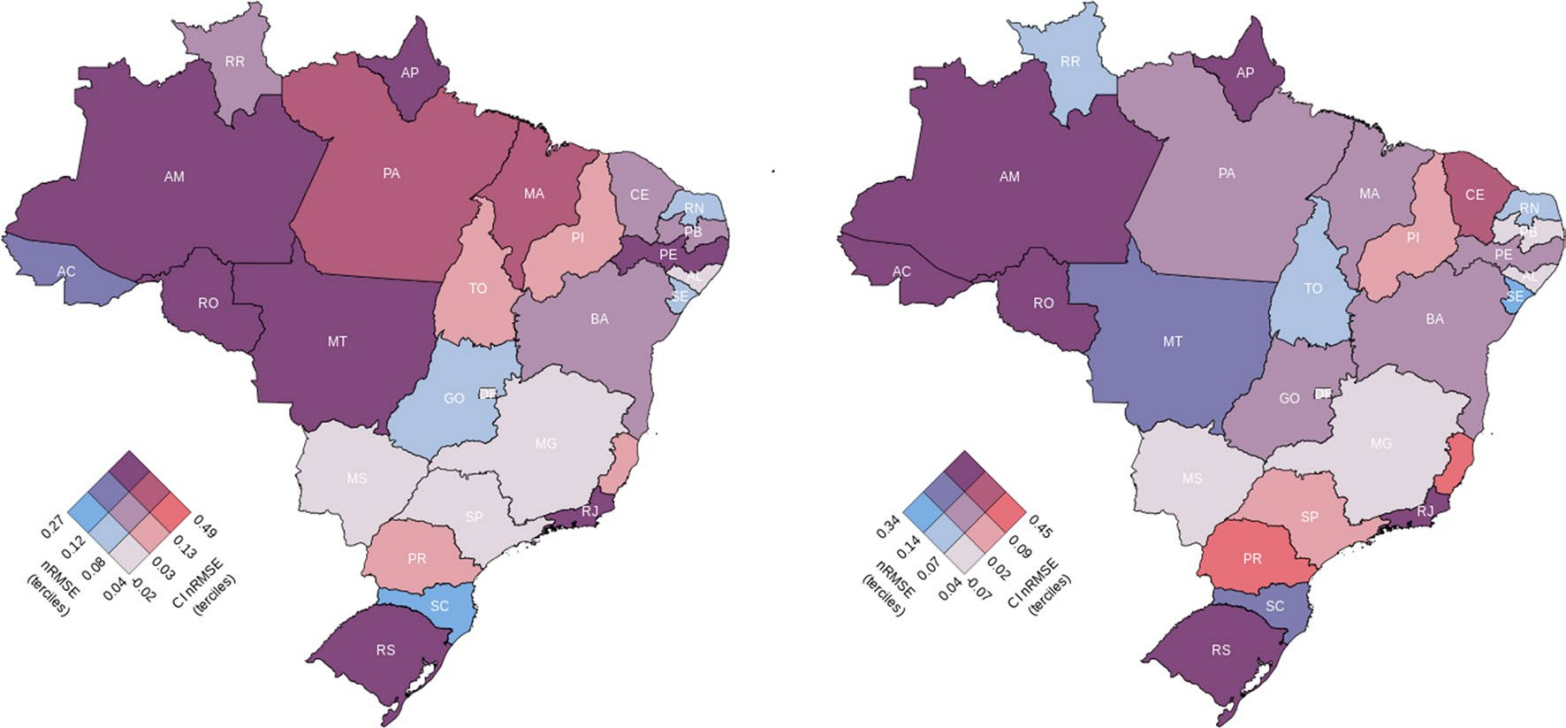
Bivariate choropleth maps displaying the nRMSE of the emsemble model distributed over the FUs, including also the nRMSE of the uncertainty (95% CI nRMSE) in its representation. Terciles divide the nRMSE and 95% CI nRMSE into three categories. Each square in the 3$\times$3 colour grid is an average of blue, representing nRMSE, and red, representing 95% CI nRMSE. (**Left**) Map for total population. (**Right**) Map for the 0–19 population group. Light purple represents lower nRMSE values with lower uncertainty, dark purple represents higher uncertainty and nRMSE values. Blue represents high nRMSE values with low uncertainty, while orange represents the opposite.

Figure 4 illustrates two bivariate choropleth maps displaying the nRMSE of the ensemble model as well as the nRMSE of its confidence interval (95% CI) for the validation dataset across all FUs, for the total population and the 0–19 population groups, respectively. As demonstrated, the ensemble model behaves better, i.e. yields lower nRMSE values with lower uncertainty (in light purple), for FUs such as Minas Gerais (MG) and Mato Grosso do Sul (MS), while higher uncertainty and nRMSE values are displayed in FUs such as Amapá (AP) and Rondônia (RO) (in dark purple). This is due to the fact that MG and MS both exhibit a more stable seasonality on reported dengue cases over the time window considered in this study, whereas AP and RO show abrupt changes, irrespective of season, hampering the model’s ability to learn intrinsic behaviours.

**Figure 5:**
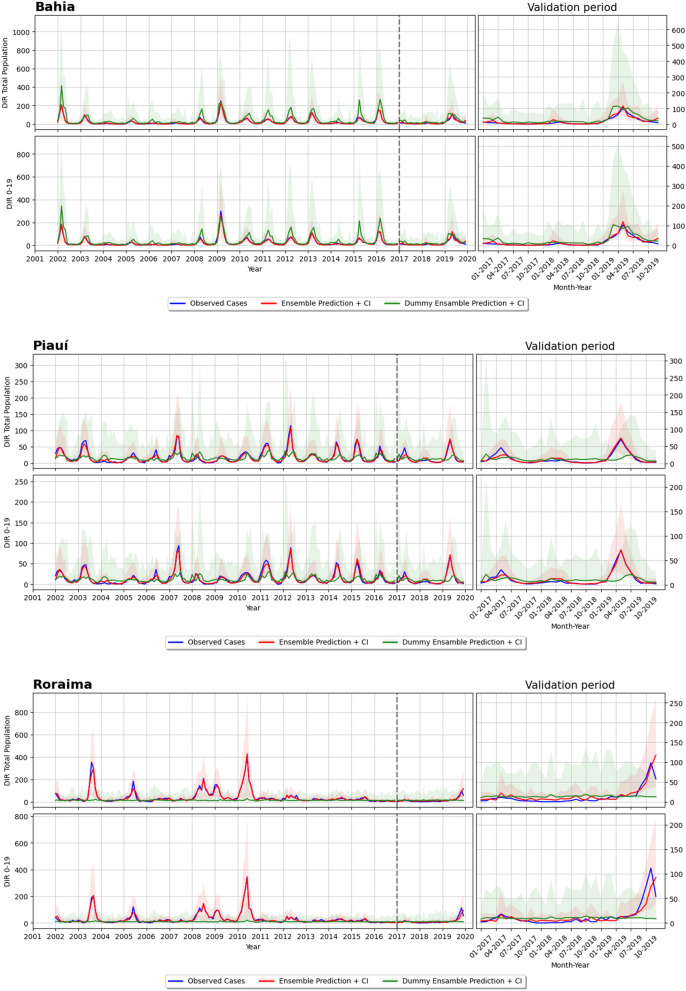
Forecasting results of the dummy and ensemble model: (**a**) Bahia, (**b**) Piauí and (**c**) Roraima. Note that these plots are structured in the save way. For each FU there are two rows, the first one for total population, while the second one for 0–19 years old group. Within each row, there are two plots: the left one shows the ground truth (or observed cases) in yellow, the ensemble prediction in red (with confidence interval) and the dummy ensemble prediction in green (with confidence interval) for the training period (2001–2016) and the validation period (2017–2019) (note that these two period are divided by a vertical dotted line); the right plot shows a zoom over the validation period.

**Figure 6:**
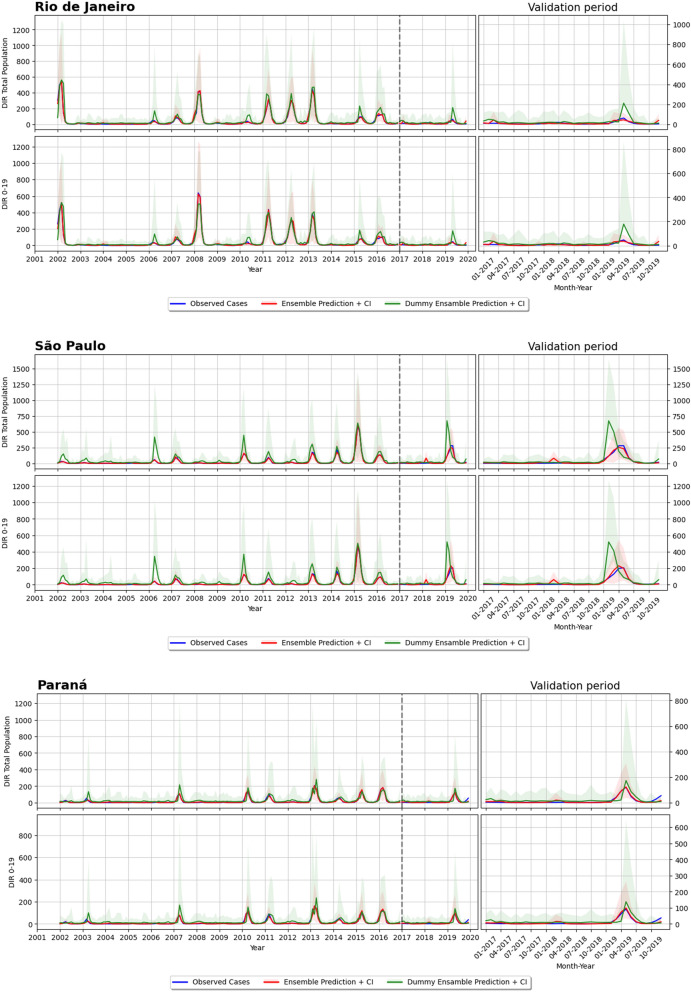
Forecasting results of the dummy and ensemble model: (**a**) Rio de Janeiro, (**b**) Sao Paulo and (**c**) Parana. Note that these plots are structured in the save way. For each FU there are two rows, the first one for total population, while the second one for 0–19 years old group. Within each row, there are two plots: the left one shows the ground truth (or observed cases) in yellow, the ensemble prediction in red (with confidence interval) and the dummy ensemble prediction in green (with confidence interval) for the training period (2001–2016) and the validation period (2017–2019) (note that these two period are divided by a vertical dotted line); the right plot shows a zoom over the validation period.

Figures 5 and 6 display typical situations that were found while observing the results on forecasting DIR for the total population, both on the training set (2001–2016) and the validation set (2017–2019). A black-dashed vertical line divides the left graph into two results parts: training and validation intervals. The graph on the right is a zoom on the validation years. The plots show the observed cases (ground truth) in yellow and the results of the ensemble model (and 95% CI) in red. To be noted that the y-axis scale changes according to the proportion of the DIR verified in each FU. The proposed model follows the seasonality pattern and the peaks of epidemics, as in São Paulo and Piauí. In Rio de Janeiro, the ensemble failed to follow the DIR behaviour, overestimating its value in 2017 and underestimating both 2018 and 2019 peaks. However, taking into account the y-axis scale, the error made by the ensemble in this example is resonable.

### Impact of Earth observation and other geospatial variables

We tested our ensemble model by removing all the ancillary variables from the inputs while keeping the DIR in the previous months. As expected, this new model, here defined as the “dummy ensemble”, always fails in forecasting DIR. This happens not only on the validation period, but also during the training one, demonstrating that EO data, as well as epidemiological and socio-economical data, is playing an important role in making the models more roboust and able to correctly forecast DIR. Visual results for this model are reported in Figs. 5 and 6. These plots shows that the model has a much higher confidence interval than the ensemble model, a tendency to overestimating or underestimating DIR (based on the FU under analysis) and in some cases shows a constant bias.

### Ablation study

We conducted an ablation study to compare the performance of the ensemble model with the single models that compose the ensemble, in order to measure the impact of these components on the overall performance of the ensemble. Essentially, we tested intermediate models on the same validation dataset, and we reported the results in Table 3. As expected, we can see that the ensemble architecture performs better than the single components in most of the cases. However, it is noteworthy that CatBoost, SVM and LSTM models behave differently across FUs, i.e. there is not one predominant single-model in terms of performance. As a result, this is an indication that all three input models contribute in a similar way to improve the inference ability of the ensemble model.

**Table 3:** nRMSE values on validation dataset for the single models. Best solution is represented in bold.

Code	Federal Unit (FU)	Ensemble		CatBoost		SVM		LSTM	
		All	0–19	All	0–19	All	0–19	All	0–19
11	Rondônia (RO)	0.237	0.216	0.301	0.259	0.357	0.310	**0.170**	**0.150**
12	Acre (AC)	0.274	0.343	0.235	0.278	**0.169**	**0.163**	0.443	0.507
13	Amazonas (AM)	0.225	0.260	0.169	**0.194**	**0.168**	0.209	0.204	0.231
14	Roraima (RR)	**0.120**	**0.104**	0.150	0.132	0.133	0.126	0.160	0.169
15	Pará (PA)	**0.123**	**0.117**	0.131	0.135	0.192	0.172	0.159	0.173
16	Amapá (AP)	**0.210**	0.207	0.248	**0.171**	0.387	0.311	0.330	0.288
17	Tocantins (TO)	**0.074**	**0.074**	0.090	0.091	0.108	0.117	0.168	0.181
21	Maranhão (MA)	**0.107**	**0.129**	0.164	0.174	0.186	0.210	0.159	0.197
22	Piauí (PI)	**0.075**	**0.046**	0.098	0.101	0.115	0.115	0.160	0.159
23	Ceará (CE)	0.080	0.087	**0.077**	**0.085**	0.108	0.131	0.109	0.104
24	Rio Grande do Norte (RN)	**0.106**	**0.123**	0.125	0.149	0.143	0.177	0.191	0.262
25	Paraíba (PB)	**0.079**	**0.051**	0.087	0.114	0.108	0.128	0.151	0.180
26	Pernambuco (PE)	0.125	**0.091**	0.151	0.116	0.145	0.120	**0.110**	0.152
27	Alagoas (AL)	**0.052**	**0.059**	0.133	0.137	0.132	0.139	0.182	0.194
28	Sergipe (SE)	**0.113**	0.154	0.157	**0.138**	0.135	0.153	0.148	0.186
29	Bahia (BA)	**0.083**	**0.081**	0.095	0.098	0.128	0.116	0.149	0.163
31	Minas Gerais (MG)	**0.050**	**0.050**	0.110	0.110	0.117	0.119	0.163	0.160
32	Espírito Santo (ES)	**0.057**	**0.062**	0.121	0.100	0.110	0.104	0.167	0.158
33	Rio de Janeiro (RJ)	0.162	0.171	**0.151**	0.169	0.260	0.314	0.155	**0.149**
35	São Paulo (SP)	**0.064**	**0.071**	0.099	0.084	0.124	0.115	0.163	0.143
41	Paraná (PR)	**0.076**	**0.071**	0.119	0.105	0.167	0.158	0.167	0.152
42	Santa Catarina (SC)	**0.220**	**0.200**	0.260	0.260	0.716	0.773	0.237	0.225
43	Rio Grande do Sul (RS)	0.227	0.222	0.385	0.440	0.857	1.021	**0.216**	**0.209**
50	Mato Grosso do Sul (MS)	**0.060**	**0.067**	0.103	0.108	0.109	0.115	0.175	0.178
51	Mato Grosso (MT)	0.218	0.169	0.194	**0.167**	0.262	0.178	**0.173**	0.188
52	Goiás (GO)	**0.087**	0.097	0.110	0.100	0.098	**0.084**	0.105	0.101
53	Distrito Federal (DF)	**0.041**	**0.040**	0.117	0.107	0.121	0.116	0.179	0.178

### Transfer learning: Peru

**Figure 7:**
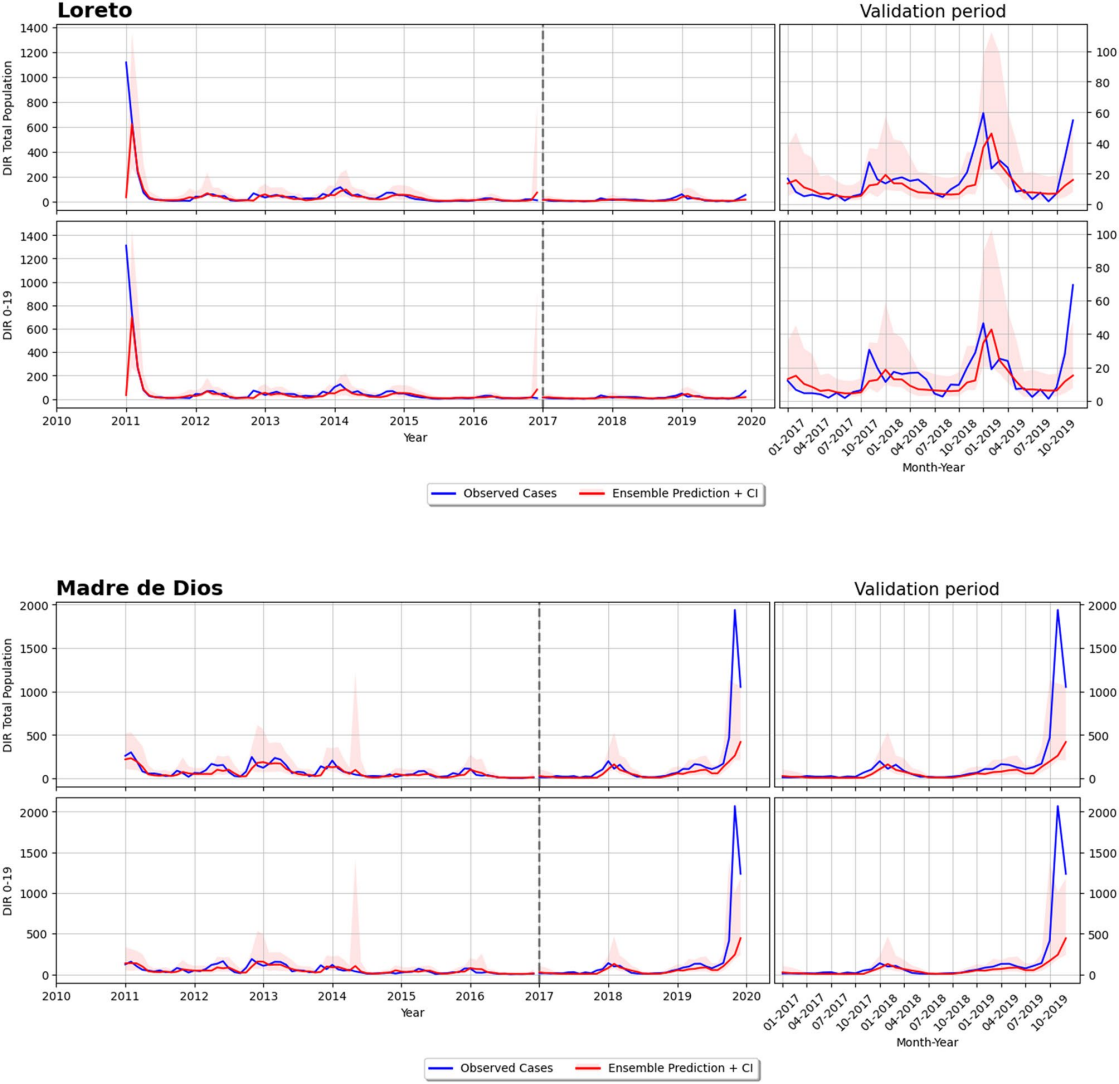
Forecasting results of the ensemble: (**a**) Loreto and (**b**) Madre de Dios. Note that these plots are structured in the save way. For each FU there are two rows, the first one for total population, while the second one for 0–19 years old group. Within each row, there are two plots: the left one shows the ground truth (or observed cases) in yellow and the ensemble prediction in red (with confidence interval) for the training period (2010–2016) and the validation period (2017–2019) (note that these two period are divided by a vertical dotted line); the right plot shows a zoom over the validation period.

In this section we tested the ensemble model on a different geographical area: Peru. We collected all data to build the Peruvian dataset but some variables, mainly from socio-economic data, are different from the Brazilian data. For this reason we fine-tuned the ensemble model to slightly adapt to these differences. As reported for the Brazilian FUs, we also report the nRMSE for some departments of Peru (Table 4). The ensemble method demonstrated a good generalisation capacity as it was able to work in other geographical area based on a different dataset. It is important to mention that even dealing with a much smaller dataset, the fine-tuning procedure was useful for a moderate improvement of the performance. To be noted that we did not run the ensemble model for all departments in Peru due to the lack of recorded dengue cases. Figure 7 illustrates the graphical results for two Peruvian departments: Loreto and Madre de Dios.

**Table 4:** nRMSE values on the validation dataset for Peru.

Department	Ensemble	
	All	0–19
Loreto	0.190	0.177
Madre de Dios	0.157	0.162
Piura	0.117	0.097
San Martin	0.160	0.138
Tumbes	0.156	0.162
Ucayali	0.294	0.327

## Discussion

An ensemble of ML models is an effective strategy to model complex regression tasks that usually rely on a single-learner architecture. In this study, we aimed to build an ensemble ML model capable of forecasting DIR one month ahead. Brazil was the main study area, from where the dataset was used to create a transferable model, later tested in Peru. The results from both countries clearly demonstrated the effectiveness of our framework, which can be transformed in an operational service, therefore guiding national governments on when to respond to a potential dengue outbreak.

The ensemble ML strategy has been compared to a model trained without EO, epidemiological and socio-economical data, proving that the former outperforms the latter in all the federal units of Brazil. The results confirm that ensemble approaches together with a multivariable dataset can provide relevant results when complex scenarios are considered. Indeed, the only time-history of dengue in previous years is not enough to provide operative and efficient prediction of future cases, as the disease is strongly affected by several external factors, such as climate change and population distribution.

This is later on confirmed by the DIR forecasting results presented in Figs. 5, 6 and 7, which demonstrated the ability of the proposed model in most Brazilian FUs and Peruvian departments. This good predictability can be appreciated quite well in the outbreak happening in 2019 for all the six reported FUs of Brazil, where the peaks of the observed cases (green curve) are properly followed by the ensemble prediction (red curve). Furthermore, the predictions of the ensemble model have generally lower uncertainty compared to the other mode, as can be seen by their 95% CI over the validation period. The small confidence interval is another advantage of the proposed ensemble model, as it guarantees that the model inferences are reliable and do not diverge too much from the observed cases.

The generalisation ability of the ensemble model is still limited for those regions reporting DIR values outside the main behaviour found in the training set. In case of FUs with DIR close to zero, the model was not able to provide an optimal performance. Nonetheless, this limitation in dealing with extreme values is a well-known ML disadvantage and well reported in the literature^58^; therefore, it does not represent a specific problem in our application.

Regarding the features and the advantages of the ensemble model, the contribution of each single ML regressor has been discussed, proving that the final random forest can take advantage of the complementary inference abilities of the input models. The LSTM, for instance, struggled with low cases but provided good performance for high cases, whereas CatBoost struggled with high cases but provided good performance for low cases. Combining these complementary abilities together allows the ensemble model to weight differently the predictions of each sub-model in order to perform under the best possible conditions in all the possible scenarios.

When considering the transferability of the ML model and the consistency of the collected input data in different states, it is worth mentioning that social variables can vary among different national census and we can expect differences in the input data bringing some problems in generalising our approach. Fortunately, the data reduction pre-processing (which maps the input dataset into another, reduced dataset) along with the fine-tuning^59^ of the ML framework can help with transferring the knowledge from one state to another. It should also be considered that if the data pre-processing is not sufficient to deal with input data from new countries, further improvements can be achieved by performing a fine-tuning training step in the ensemble model to cope with the new content and distribution of data. The generalisation ability of the model can be enforced also in the design and conceptualisation of the machine learning approach. For instance, choosing relatively simple training loss functions prevents the overall model from being too specific for a single training area, i.e., it prevents overfitting on the training data.

This study also faced some limitations that must be acknowledged. First, to make our trained ensemble ML model efficient and transferable, the same (or similar) list of variables must be collected in the new location. Unfortunately, extracting long time-series data (i.e., 20 years of DIR for Brazil) is not possible in other countries. This is why we demonstrated how we transferred our ensemble model to Peru, which had half of this temporal length and administrative units with available data. Second, we did not work with all departments in Peru; therefore, it was not feasible to compute the spatial correlation through the CNN-based pre-processing step. However, we solved this limitation by bypassing this step. Third, the Brazilian DIR data was a value epidemiological dataset to train and validate our ensemble model, since the DIR distribution is very heterogeneous across FUs. However, this diversity in DIR behaviour across FUs is also the reason why we developed an ensemble ML model. The ability of the ensemble model to appropriately detect the DIR temporal signature is strongly related to the combination of different single-ML models, (i.e. SVM, LSTM, and CatBoost). Each single-ML model had its own strengths and limitations which, once combined, can be exploited to get the overall maximum performance.

To summarise, the core of our innovation lies not only within the algorithms themselves, but in their application to a domain fraught with data scarcity and the need for operational scalability. Our work distinguishes itself by leveraging well-curated ground data, meticulously collected from actual field operations, and marrying it with academic research to devise methodologies that are both practical and scalable. This fusion of rigorous on-the-ground data collection with advanced analytical methods is rare and addresses a significant gap in current practices.

To elucidate the gravity of overcoming the dearth of real-world data we pose a reflective question: How often are these novel technologies seen actively integrated into the workflows of bureaucratic organisations, such as those within the UN? The answer, we find, is not often, which signals the groundbreaking nature of our efforts. The challenge of starting such an approach is twofold: first, it requires the establishment of a robust data foundation which is often absent, particularly in long-term datasets. Second, it necessitates a paradigm shift within these organisations towards a more data-centric approach in operational methodology. Our project serves as a testament to overcoming these hurdles.

In sum, our work provides a solid template for how academic research can effectively inform and enhance practical applications, even within traditionally slow-to-adapt structures.

## Conclusion

In summary, our study utilized an ensemble machine learning framework for one-month-ahead Dengue Incidence Rate (DIR) forecasting in large administrative areas of Brazil and departments in Peru. We combined data from multiple sources, including earth observation satellite products, climate reanalysis models, social-economic variables, and geospatial features for the period of 2001–2019. The ensemble model showed good accuracy across the Brazilian FUs and an excellent performance in transferring the approach to another country, even under the explained constraints found in Peru.

Results, especially during the 2019 outbreak in Brazil, highlighted the ensemble model’s ability to predict DIR trends with low uncertainty. Despite acknowledged limitations in handling extreme values, our approach excelled in regions with recurrent epidemic outbreaks.

We discussed the transferability of the model, addressing variations in social variables and showcasing successful adaptation to Peru. Our work not only advances DIR forecasting but also represents a practical shift in integrating advanced analytics into public health operational frameworks. The study’s success in diverse settings underscores its scalability and sets a precedent for collaborative interdisciplinary efforts in addressing global health challenges. Moreover, the transferability of the trained model to another location is our main goal and a great contribution to the scientific community and to UNICEF, who is working closer with national governments to replicate this framework. In this work, we developed a methodology that can be easily applied to other locations also affected by dengue epidemics.

The collaborative nature of this work, involving intergovernmental organizations and public health institutions, emphasizes the importance of complementary expertise in advancing knowledge and addressing societal challenges. Essentially, our research contributes to understanding the factors behind dengue outbreaks and encourages interdisciplinary collaboration and the practical use of advanced analytical methods in public health.

## Appendix 1: Socio-economic variables description

The intent of this appendix section is to report the socio-economic variables used to build our dataset. To lighten the discussion of the main body, we preferred to list them here, in Table 5.

**Table 5:** List of Brazilian socio-economic variables used in this case study. Refer to^47^ for further details.

Socio-economic variables	
Index of Social Vulnerability (ISV). Arithmetic average between three dimensions: ISV Urban Infrastructure, ISV Human Capital, ISV Income and Work.	
Index of Urban Infrastructure.	
Index Human Capital.	
Index Income and Work.	
Ratio of people living in permanent private households with inadequate water supply and sanitation.	
Ratio of people living in permanent private households with electric lighting.	
Ratio of people living in permanent private urban households without garbage collection service.	
Ratio of children living in households where none of the residents have completed elementary school.	
Ratio of people aged between 15 and 24 years who do not study nor work and have a per capita household income equal to or less than half the minimum wage (of 2010).	
Ratio of people living in households with per capita income less than half the minimum wage (of 2010).	
Ratio of people living in households with per capita income less than half the minimum wage (of 2010) that spend more than one hour commuting.	
Unemployment rate of the population aged 18 years and over.	
Ratio of people aged 18 years or over without complete elementary school and unofficially employed.	
Index of Municipal Human Development (IMHD). Arithmetic average between three dimensions: Income, Education and Longevity indices.	
Index of Longevity.	
Index of Education.	
Index of Income.	
Sub-index selected to compose the IMHD Education, representing the education level of the adult population.	
Sub-index selected to compose the IMHD Education, representing the attendance of children and young people to school in grades appropriate to their age.	
Ratio of people aged 18 years and over with complete elementary school.	
Per capita income.	
Per capita income of the people vulnerable to poverty.	
Economically active population aged between 10 and 14 years.	
Economically active population aged between 15 and 17 years.	
Economically active population aged 18 years and over.	
Illiteracy rate of the population aged 15 years and over.	
Illiteracy rate of the population aged 18 years and over.	
Ratio of people officially employed aged 18 years and over.	
Ratio of people officially employed with complete elementary school aged 18 years and over.	
Ratio of people officially employed with complete middle school aged 18 years and over.	
Ratio of people living in permanent private households with a density of residents greater than 2.	
